# Husband and wife with sarcoidosis: possible environmental factors involved

**DOI:** 10.1186/2049-6958-8-5

**Published:** 2013-01-25

**Authors:** Ilaria Leli, Ivano Salimbene, Francesco Varone, Leonello Fuso, Salvatore Valente

**Affiliations:** 1 Department of Pulmonary Medicine, A. Gemelli University Polyclinic, Sacro Cuore Catholic University, Largo A. Gemelli 8, Rome, 00168, Italy

**Keywords:** Environmental factors, Granuloma, Sarcoidosis

## Abstract

Sarcoidosis is a granulomatous multisystem disorder of unclear etiology that involves any organ, most commonly the lung and the lymph nodes. It is hypothesized that the disease derives from the interaction between single or multiple environmental factors and genetically determined host factors. Multiple potential etiologic agents for sarcoidosis have been proposed without any definitive demonstration of causality.

We report the case of two patients, husband (57 years old) and wife (55 years old), both suffering from sarcoidosis. They underwent a lymph node biopsy by mediastinoscopy which showed a “granulomatous epithelioid giant cell non-necrotising chronic lymphadenitis”. They had lived up to 3 years ago in the country in a farm, in contact with organic dusts, animals such as dogs, chickens, rabbits, pigeons; now they have lived since about 3 years in an urban area where there are numerous chemical industries and stone quarries. The aim of this case report was to focus on environmental factors that might be related to the pathogenesis of the sarcoidosis.

## Background

Sarcoidosis is a granulomatous multisystem disorder of unclear etiology that involves any organ, most commonly the lung and the intrathoracic lymph nodes. Also heart, skin, and central nervous system are frequently affected. The diagnosis of sarcoidosis is further hindered by the lack of any reliable and specific diagnostic test, as there are no laboratory or imaging findings that enable the definitive diagnosis. Even the histological data are not conclusive because the non-necrotising granuloma, a hallmark of morphologic appearance of the disease, is not unique for sarcoidosis. Granulomas typically consist of a compact central area of macrophages that differentiate into epithelioid cells and then fuse to form multinucleated giant cells surrounded by lymphocytes.

A genetic predisposition to sarcoidosis is epidemiologically evidenced by the demonstration of familial aggregation, differences in disease susceptibility and severity between racial groups, and the significantly increased incidence in monozygotic twins of affected individuals compared to other siblings [1,2]. It is hypothesized that the disease derives from the interaction between single or multiple environmental factors and genetically determined host factors. Multiple potential etiologic agents for sarcoidosis have been proposed without any definitive demonstration of causality. However, environmental causes of sarcoidosis or sarcoidosis-like granulomatous diseases are well established, especially after industrial exposure to beryllium [3]. An enduring hypothesis is that based on an infectious etiology, with *Mycobacterium spp*. being the most commonly implicated organism. The role of various non-infectious organic and inorganic environmental agents has been studied in the pathogenesis of sarcoidosis.

## Case presentation

We report the case of two patients, husband and wife who had lived up to 3 years ago in the open country, in contact with organic dusts (hay and molds), and animals such as dogs, chickens, rabbits, pigeons; now they live in an urban area where there are numerous chemicals industries and stone quarries.

**Figure 1:**
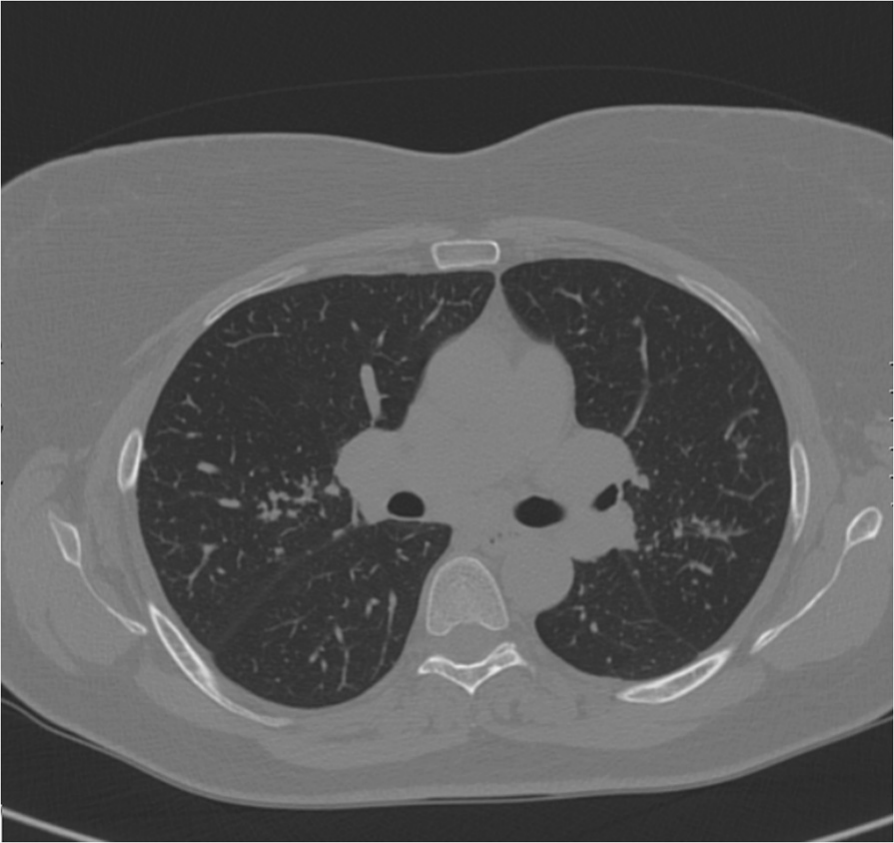
Chest CT scan of the woman showing: “a series of lung nodules distributed in all lobes”.

The woman, 55 years old, is a housewife and a former smoker of 1 pack/years. She underwent a hysterectomy for uterine fibromatosis, and a surgical operation for right humerus osteochondroma. She has got a herpes simplex virus type I infection. At the time of our first observation she referred in the last 2 years dry cough with little sputum, dyspnea grade II according to the American Thoracic Society (ATS) scale and mild fever. At the beginning of the symptoms she performed a chest x-ray which showed “interstitial thickening” and a microbiological sputum examination which was negative for tuberculosis and fungal infection. The general practitioner gave her a non-specific antibiotic therapy with low benefit and then a steroid therapy with improvement in symptoms. At the time of our observation, she underwent a total-body CT scan which showed “a series of lung nodules distributed in all lobes; a solid slab in the mantle of the apical segment of the left lower lobe; several small adenomegalies in the anterior mediastinum, around the trachea and below aorta artery; no evidence of effusion in the pleural cavity” in the thorax (Figure 1). The CT scan showed the “presence of enlarged lymph nodes in the following areas: celiac/pancreatic lombo-aortical, bilateral internal iliac, bilateral obturator, bilateral external iliac and bilateral inguinal” in the abdomen.

The 6-min walking test was normal and the pulmonary function tests showed a reduction in the diffusing capacity for carbon monoxide (DL_CO_) (Table 1). The arterial blood gas analysis performed during spontaneous breathing at FiO_2_ of 0.21 showed mild hypoxemia at rest with minimal alterations of intrapulmonary gas exchange, and normal acid–base balance: PaO_2:_ 73 mmHg; PaCO_2:_ 37,9 mmHg; pH: 7,41. The serum level of angiotensin I-converting enzyme (ACE) was normal.

**Table 1:** Respiratory function results in the woman

	
VC (% pred)	109
FEV_1_ (% pred)	106
FEV_1_/FVC (%)	102
TLC (% pred)	98
RV (% pred)	89
DL_CO_ (% pred)	58

DL_CO,_ Lung diffusing capacity for carbon monoxide. FEV_1_, Forced expiratory volume in1 sec; FVC, Forced vital capacity; RV, Residual volume; TLC, Total lung capacity; VC, Vital capacity.

The man, 57 years old, truck driver, is a former smoker of 54 pack/years. He has arterial hypertension, type 2 diabetes mellitus, chronic renal failure with mild anemia and an obstructive sleep apnea syndrome (OSAS) in severe obesity. As his wife, he has got a herpes simplex virus type I infection. He takes regular therapy with aspirin, doxazosin, allopurinol, irbesartan, erythropoietin and insulin. He referred dyspnea ATS grade II-III in the absence of cough. He also reported to have taken steroids in the past at the starting dose of 50 mg/day and gradually tapered. This therapy was recommended in the absence of a definitive diagnosis and on the basis of symptoms and radiological abnormalities of “interstitial thickening” present in the chest x-ray. At the time of our first observation, the pulmonary function tests revealed a mild restrictive pattern with reduction of DL_CO_ (Table 2). The 6-min walking test showed that the distance walked during sub-maximal exercise was below the lower normal limit. The high-resolution chest CT scan showed “areas of increased ground-glass density in the right lower lobe, with parenchymal micro-nodules in the following lobes: left upper, lingula, left lower, middle, and right lower; multiple enlarged lymph nodes are present in the mediastinum, at the following areas: retrotracheal, paratracheal, aortopulmonary window, subcarinal and bilateral hilar; other enlarged lymph nodes are present at the origin of neck vessels, in the supraclavicular zone, and (in scans passing through the upper abdomen) bilaterally along the celiac tripod, peripancreatic area, along the gastro-lienal and epatogastric ligament”. Both patients underwent bronchoscopy with bronchoalveolar lavage (BAL) and transbronchial biopsy (TBB). The BAL fluid revealed a mild lymphocytic alveolitis both in the man (L = 22%) and in the woman (L = 18%) with a CD4/CD8 ratio of 3.1 in the former and 2.8 in the latter. The microbiological analysis of the BAL fluid was negative for common bacterial, tubercular and fungal infections. The TBB was non-diagnosticboth in man and in woman due to a poor and non-representative lung sample. For these reasons both patients underwent lymph node biopsy by mediastinoscopy which showed a “granulomatous epithelioid giant cell non-necrotising chronic lymphadenitis”.

**Table 2:** Respiratory function results in the man

	
VC (% pred)	75
FEV_1_ (% pred)	82
FEV_1_/FVC (%)	82
TLC (% pred)	75
RV (% pred)	80
DL_CO_ (% pred)	59

DL_CO_, Lung diffusing capacity for carbon monoxide; FEV_1_, Forced expiratory volume in1 sec; FVC, Forced vital capacity; RV, Residual volume; TLC, Total lung capacity; VC, Vital capacity.

## Discussion

The environmental exposition of the two patients concerns chemical agents because of the industries near where they lived in the last years and because of the presence of a sand quarry. The industries in the area where they live include those involved in the processing of tires. Thus, factors such as the inorganic powder used in the rubber industry can play an active role in the pathogenesis of their disease. Other factors potentially involved could be silica, silicone and the fibers of the glass. Three recent longitudinal studies regarding the World Trade Center disaster have emphasized the role of airborne inorganic particulate exposure in sarcoidosis [4-6]. These studies showed an increase in sarcoidosis among firefighters and emergency first responders. Jordan et al. [4] followed World Trade Center health registry participants, including 43 patients with sarcoidosis confirmed on clinical and histological basis. They stratified individuals in their cohort based on self-reported dust cloud exposures and observed that a more intensive rescue and recovery activities was related to an increased risk of disease [5]. Additionally, Crowley et al. [6] calculated an extraordinarily high annual incidence rate of 229 cases of sarcoidosis per 100,000 person-years, based on a record review of nearly 20,000 individuals in the World Trade Center medical surveillance program. The World Trade Center dust was a highly alkaline mixture that the US Geological Survey characterized as containing mostly the components of concrete, including gypsum, anhydrite, and manmade vitreous fibers. These epidemiological studies do not demonstrate the components or the mechanisms by which this dust triggers granuloma formation. However, taken as a whole, this body of literature on World Trade Center sarcoidosis helps to validate the case series and case reports of the last decades reporting sarcoidosis occurring after exposure to silica, talc, and other inorganic triggers.

Geographic clustering of disease in the southern United States and in other parts of the world has promoted further speculations concerning weather, soil, and foliage in the pathogenesis of sarcoidosis [7]. Past studies have noted high prevalence of sarcoidosis where there is lumbering activity and exposure to farm animals and pets [8]. Rural residence, birthplace or time spent in rural regions have been associated with sarcoidosis [7]. Cummings et al. [9] observed clustering in communities with lumbering or wood milling as the principal local industry.

Interestingly, other studies suggest the hypothesis of either a person-to-person transmission or a shared exposure to an environmental agent. In a well documented clustering that has occurred in the relatively isolated population of the Isle of Man, individuals with sarcoidosis were more likely to have had prior contact with an individual who had sarcoidosis, compared to controls (39.6% versus 1.1%). These contacts included neighbors, unrelated cohabitants, and co-workers [10]. Similarly, there has been a cluster of cases among sisters and unrelated social contacts, including one sister’s employer [11]. Although familial clustering may be explained on the basis of shared genes, shared common environmental exposures must be considered as well.

Finally, several observations suggest that sarcoidosis may be caused by an infectious agent. There are many known infectious agents that can cause granulomas that resemble those of sarcoidosis, including mycobacteria, herpes viruses, histoplasmosis, treponematosis, sporotrichosis, coccidioidomycosis, schistosomiasis, listeria, the agent of Whipple’s disease and *Rhodococcussp*[12]. Because sarcoidosis has a world-wide distribution, it is necessary to postulate that the putative agent that causes sarcoidosis is also widely distributed. However, it is also possible that sarcoidosis may be the result of several different infections. A real problem in identifying an etiologic agent causing sarcoidosis is its wide variety of clinical manifestations, which derives from the complex immunologic processes underlying the disease.

Both our patients have got labial herpes simplex virus and this could suggest a potential role of the infection in the development of the disease. Moreover, before moving to the urban area, the two patients lived in the countryside, reporting exposure to molds and hay. Finally, they were exposed to chemical agents derived from the industry of the tires. Among those, silica is a mix of modern tires and it is known that silica could be potentially involved in the pathogenesis of the disease. However, all the environmental factors that could be considered potential triggers of the disease are not sufficient by themselves to cause sarcoidosis and must interact with a particular immune response of the host. Therefore, the genetic predisposition of the patients plays a key role in the development and progression of the disease.

## Conclusions

In conclusion, this case report wants to focus on environmental factors that may be potential causative agents for sarcoidosis. Important tool in investigating patients with sarcoidosis is a complete medical history that includes occupational and environmental factors, lifestyle habits and any previous infection. It could be also useful to study and monitor the areas with the highest incidence of this disease.

## Consent

Written informed consent was obtained from the patient for publication of this Case report and any accompanying images. A copy of the written consent is available for review by the Editor-in-Chief of this journal.

## Competing interests

The authors declare that they have no competing interests.

## Authors’ contributions

IL and IS made substantial contributions to conception and design, or acquisition of data, or analysis and interpretation of data; LF and FV were involved in drafting the manuscript or revising it critically for important intellectual content; SV gave final approval of the version to be published.
